# Trends in local newspaper reporting of London cyclist fatalities 1992-2012: the role of the media in shaping the systems dynamics of cycling

**DOI:** 10.1016/j.aap.2015.10.016

**Published:** 2016-01

**Authors:** Alex Macmillan, Alex Roberts, James Woodcock, Rachel Aldred, Anna Goodman

**Affiliations:** a Department of Preventive and Social Medicine, University of Otago, Dunedin, New Zealand; b College of Medical and Dental Sciences; University of Birmingham, Birmingham, UK; c UK CRC Centre for Diet and Activity Research (CEDAR), MRC Epidemiology Unit, University of Cambridge School of Clinical Medicine, University of Cambridge, Cambridge, UK; d Department of Planning and Transport, Faculty of Architecture and the Built Environment, University of Westminster, London, UK; e Faculty of Epidemiology and Population Health, London School of Hygiene and Tropical Medicine, London, UK

**Keywords:** CI, confidence interval, Cycling, Fatality, Injury, Media, Systems dynamics, Trends

## Abstract

**Background:**

Successfully increasing cycling across a broad range of the population would confer important health benefits, but many potential cyclists are deterred by fears about traffic danger. Media coverage of road traffic crashes may reinforce this perception. As part of a wider effort to model the system dynamics of urban cycling, in this paper we examined how media coverage of cyclist fatalities in London changed across a period when the prevalence of cycling doubled. We compared this with changes in the coverage of motorcyclist fatalities as a control group.

**Methods:**

Police records of traffic crashes (STATS19) were used to identify all cyclist and motorcyclist fatalities in London between 1992 and 2012. We searched electronic archives of London's largest local newspaper to identify relevant articles (January 1992–April 2014), and sought to identify which police-reported fatalities received any media coverage. We repeated this in three smaller English cities.

**Results:**

Across the period when cycling trips doubled in London, the proportion of fatalities covered in the local media increased from 6% in 1992–1994 to 75% in 2010–2012. By contrast, the coverage of motorcyclist fatalities remained low (4% in 1992–1994 versus 5% in 2010–2012; *p* = 0.007 for interaction between mode and time period). Comparisons with other English cities suggested that the changes observed in London might not occur in smaller cities with lower absolute numbers of crashes, as in these settings fatalities are almost always covered regardless of mode share (79–100% coverage for both cyclist and motorcyclist fatalities).

**Conclusion:**

In large cities, an increase in the popularity (and therefore ‘newsworthiness’) of cycling may increase the propensity of the media to cover cyclist fatalities. This has the potential to give the public the impression that cycling has become more dangerous, and thereby initiate a negative feedback loop that dampens down further increases in cycling. Understanding these complex roles of the media in shaping cycling trends may help identify effective policy levers to achieve sustained growth in cycling.

## Highlights

• Cycling trips doubled in London between 1992 and 2012.
• The proportion of cyclist fatalities covered by media rose 13-fold over this period.
• The increased coverage was specific to cyclists, and not seen for motorcyclists.
• Such coverage may create complex feedback loops, inhibiting cycling growth.
• The relative strength of such feedback loops is likely to vary between cities.

## 1 Introduction

The health benefits of cycling are well established (Lindsay et al., 2011, Woodcock et al., 2013, Pucher et al., 2010), with the physical activity benefits substantially outweighing the injury and air pollution risks in populations where a broad range of age groups cycle (Rojas-Rueda et al., 2011, de Hartog et al., 2010, Woodcock et al., 2014). Increasing levels of cycling can also confer additional benefits including reducing urban congestion (Cabinet Office, 2009) and greenhouse gas emissions (Maizlish et al., 2013). Over the past twenty years, these benefits have prompted countries and cities across the world to develop pro-cycling policies (Aldred, 2012, Pucher et al., 2011, Butcher, 2012). This includes the recent publication of an ambitious ‘Mayor's Vision for Cycling’ in London (Greater London Authority, 2013), a city that has already seen rises in cycling. Nevertheless, cycling levels in London and other parts of the UK remain lower than those in many European countries (Gatrell, 2011). Transport for London estimates that 23% of journeys could realistically be cycled in the capital, ten times higher than at present (Transport for London, 2010). Studies examining barriers to cycling have identified multiple factors that may contribute to lack of uptake (Pooley, 2011, Steinbach et al., 2011, Gatersleben and Haddad, 2010), but one of the most common reasons people give for not cycling is perceived risk (Lawson et al., 2013, Horton, 2007, Thornton et al., 2010).

It is plausible that the media plays an important role in shaping these safety concerns (Horton, 2007). The effect of media reporting on public opinion and behaviour is widely appreciated (Kitzinger, 2007, Genovesi et al., 2010, McCombs, 2013) including evidence that media can affect road safety behaviours (Phillips et al., 2011). McCombs’ (McCombs, 2013) agenda-setting theory describes the role of the media in establishing which issues are most prominent in the public agenda (e.g. the extent to which ‘cycling’ is a topic worthy of public attention). In addition, later research also suggests that second-level agenda-setting may be at work, in defining how these issues are conceived (e.g. whether cycling is considered ‘dangerous’ or ‘trendy’) (McCombs and Stroud, 2014). This opinion-forming role may be particularly important with respect to coverage of cycling fatalities and serious injuries, because such incidents occur comparatively rarely and so are not directly experienced by most people on a regular basis. For this reason, it has been argued that people's overall perception of road traffic risks typically draws on media reporting as well as their personal perceptions of risk in their everyday lives (Hojman et al., 2005). Moreover, if the media provides memorable coverage of these comparatively rare incidents then the public may overestimate the risk of such events, a phenomenon known to psychologists as the ‘availability heuristic’ (Tversky and Kahneman, 1973). This phenomenon has been demonstrated most clearly for public fear of crime (Lowry et al., 2003, Greer, 2009). Cyclist deaths and serious injuries share aspects of newsworthiness with crime: they are easy to write about with a simple storyline and convenient access to information; have human interest to ordinary people; and may include enthralling details of violence. In addition, in the context of low levels of cycling, the absolute number of cycling deaths and injuries is low enough to permit each incident to be reported individually. This is a feature shared with aeroplane crashes, another type of risk that is overestimated by the public due to preferential media coverage (Kahneman, 2011). In this light, it is noteworthy that a recent media analysis in Australia found that the most common type of cycling-related story involved cyclists being injured (13.2%), while the second most common involved cyclists being killed (10.7%) (Rissel et al., 2010). Similar findings have been reported in London, with cycling ‘accidents and dangers’ accounting for 27% of all issues mentioned in cycling-related newspaper articles, a much higher percentage than any other category (Penalosa, 2011).

**Fig. 1:**
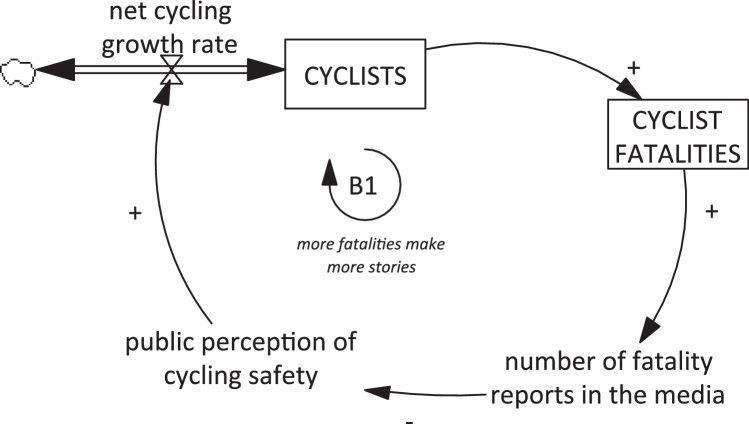
Causal loop diagram linking levels of cycling in a population and media representations of cycling. Variables in boxes are those whose levels we are interested in following over time (stocks). Arrows with a positive sign (+) indicate that a change in the arrow-tail variable leads to a change in same direction in the arrow-head variable. Arrows with a negative (−) sign indicate that a change in the arrow-tail variable leads to an inverse (opposite direction) change in the arrow-head variable. Loops labelled ‘B’ are ‘balancing loops’, which have the effect of dampening the initial pattern of behaviour.

Recently, the role of the media in shaping attitudes to cycling has attracted the attention of researchers using a ‘systems dynamics’ perspective to model the dynamic influences on cycling for transport in cities (Macmillan et al., 2014). System dynamics modelling can incorporate the complex interplay of individual, societal, environmental and policy factors shaping behaviour and synthesise these into a qualitative causal theory of positive and negative feedback loops. This dynamic causal loop diagram can then be used as the basis for quantitative simulations to inform policy (Richardson, 2011), and such approaches are increasingly applied across a range of disciplines related to safety and behaviour (Goh et al., 2014, Underwood and Waterson, 2014). In two previous pieces of research, qualitative system dynamics models exploring the determinants of trends in urban cycling have been developed through interviews and workshops with a broad range of policy, community and academic stakeholders (Macmillan et al., 2014, Macmillan and Woodcock, 2013). During this development process, many of the relationships in these earlier models have been tested through data identification and simulation (Macmillan et al., 2014). These models propose that as cycling becomes more common in a population there is also likely to be an increase in the absolute number of cycling crashes (unless this is offset by an even faster decline in the risk per cycling trip) (Macmillan and Woodcock, 2013). If the number of crashes covered by the media increases in tandem, this is likely to decrease public perceptions of cycling safety. This is particularly the case if, as has been argued by elsewhere, public perceptions of road traffic risks are more sensitive to absolute numbers of events than to changes in the underlying statistical risks per unit of travel (Hojman et al., 2005). This could, in turn, introduce a negative feedback loop, dampening the total increase in cycling levels in a balancing loop (balancing loop ‘B1’ in Fig. 1). To our knowledge, however, there exists no empirical evidence concerning this particular part of the model, namely the relationship between changes in the prevalence of cycling and changes in media coverage of cycling road traffic crashes.

**Fig. 2:**
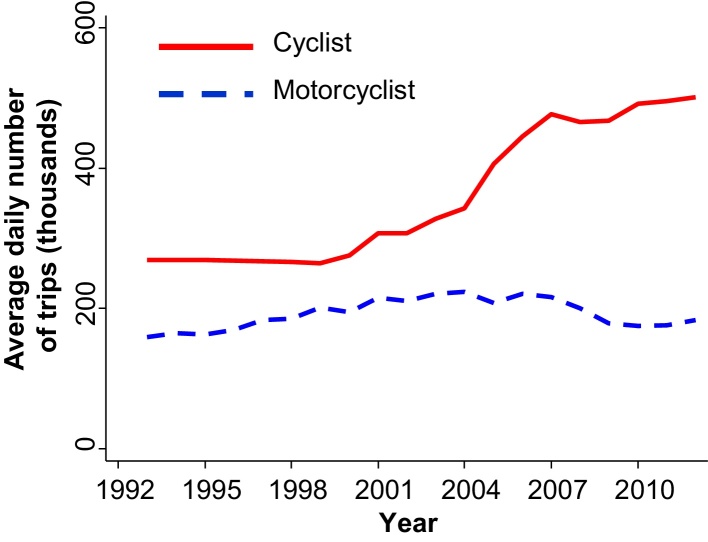
Estimated daily number of cycle and motorcycle journeys in Greater London, 1993–2012. Annual estimates published in the Transport for London Group Planning ‘Strategic Analysis’ (Transport for London, 2012b, plus subsequent personal communication from Graeme Fairnie, 26/06/2014). Data from the National Travel Survey (Department for Transport, 2013) provided no evidence that the average distance or duration per cycle trip changed between 2002 and 2012 for cycling (*p* > 0.15, based on 6694 trips) or motorcycle trips (*p* > 0.25, based on 1618 trips).

This paper therefore aimed to examine this relationship in London, a city in which cycling levels have almost doubled in the past 20 years (e.g. cycling represented 2.2% of commute trips in London in the 1991 census, 2.5% in 2001, and 4.3% in 2011 (Census, 2013); see also Fig. 2). Within the boundary of our model about urban cycling, our aim was to examine whether this change in the prevalence of cycling was associated with changes in the proportion of cyclist fatalities covered by London's largest local newspaper, and in the amount of coverage per fatality. In order to assess whether any observed changes might simply reflect wider trends in media coverage of road traffic crashes, rather than being specifically associated with increased cycling, we used the coverage of motorcyclist fatalities as a control. We used this as our control because (i) motorcycling is another a minority transport mode that carries a comparatively high risk of injuries, (ii) the prevalence of motorcycling in London remained relatively stable (1.2% commute modal share in 1991, 1.6% in 2001, 1.2% in 2011; see also Fig. 2), and (iii) pilot work indicated that motorcyclist fatalities resembled cyclist fatalities in being fairly readily identified in newspaper reports using keyword searches. We also sought to use a similar approach to compare London to three other English cities, with contrasting recent trajectories in cycling levels.

## 2 Methods

### 2.1 Police-reported road traffic fatalities

We identified cyclist and motorcyclist fatalities reported to the police, and used this as a denominator for our subsequent examination of coverage rates in the local media. We identified these denominator fatalities using the ‘STATS19’ dataset, which records details of all road traffic injuries that occur on the public highway and that are reported to the police. Comparisons between STATS19 and hospital admission data indicate that a high proportion of injuries are reported to the police in London (Ward et al., 2002), and this figure is likely to be particularly high for fatalities. In previous work we have argued that it is plausible that STATS19 covers around 95% of all cycling fatalities in London (Woodcock et al., 2014).

In London, we identified all fatalities across a 20-year period (01/01/1992–31/12/2012) in which the casualty was travelling by bicycle (*N* = 325) or by motorcycle/moped (henceforth motorcycle; *N* = 893). Data available from STATS19 on each fatality included: the date of the fatality; the location of the crash; the age and sex of the casualty; and the mode of travel of any other vehicle involved in the fatality. We used this last variable to assign a ‘strike mode’ to each fatality, defined as the largest other vehicle involved. This could include ‘no other vehicle’ for cases in which, for example, a motorcyclist lost control of his motorbike.

Ethical approval was not required as all data were fully in the public domain.

### 2.2 Matching fatalities to local newspaper coverage

When examining which of these police-reported fatalities were covered in the local media, we focussed on articles in the London Evening Standard. The Evening Standard is the most widely-distributed city-specific daily newspaper in London (Transport for London, 2012a) and its archives were accessed via the electronic journalist database Lexis Library (http://lexisnexis.com). We searched for cycling articles in the Lexis Library using the Boolean search ([‘cyclist’ OR ‘bicycle’] AND [‘died’ OR ‘death’ OR ‘killed’]), and for motorcyclist articles using the search ([‘Motorcycle’ OR ‘motorcyclist’ OR ‘motorbike’ OR ‘biker’ OR ‘moped’ OR ‘Vespa’] AND [‘died’ OR ‘death’ OR ‘killed’]). These searches were developed and refined through a piloting phase which compared the return of different searches.

We performed this search of the London Evening Standard archives across the period January 1992–December 2014, and were returned 2041 articles on the cyclist search and 1875 on the motorcyclist search. AR and/or AG then read through these to identify articles that referred to a particular cyclist or motorcyclist fatality in STATS19. Articles were linked to an individual case based on time, date, gender, age, strike mode and area. For a STATS19 fatality to count as having been ‘covered’, the individual did not have to be named but there did have to be enough information to identify the case specifically. Thus, for example, “a woman cyclist and her daughter were killed today on London Bridge” would count as coverage for the cases in question, but “7 women cyclists were killed last year in London” would not. Multiple articles could be assigned to the same individual if their fatality was covered more than once. On a sample of 70 STATS19 fatalities, inter-rater reliability between AR and AG was 97% for our primary outcome, which was whether a particular fatality received any media coverage within two years of the death.

In addition to this pre-specified data extraction, our emerging findings prompted us to conduct a post hoc analysis in which we identified all articles in which the headline described a call or campaign for cycle safety improvements (e.g. “Fiancé of cyclist killed by lorry calls for safety mirrors”) or criticised the safety of existing infrastructure (e.g. “Cyclists’ fury over killer bridge that's STILL a blackspot”). All headlines were assessed independently by both AG and RA (inter-rater agreement 96%, disagreements resolved by consensus).

In the course of searching for the 1218 London fatalities recorded in STATS19, we found newspaper reports of a further 6 fatalities (0.5%: 4 cyclist, 2 motorcyclist) which were not in STATS19, despite appearing eligible for inclusion in that database. We did not include these 6 fatalities in our analyses; sensitivity analyses indicated that this did not materially affect any of our findings.

### 2.3 Replication across other English cities

We replicated the approach described in Sections 2.1, 2.2 across three other English cities, chosen purposively to provide a mixture of contrasting cycling trajectories. The first, Birmingham, is Britain's second largest city and has experienced low levels of cycling throughout the study period (1.4% of commuters cycling to work in 1991, 1.7% in 2011). The second, Bristol, is the largest British city outside of London to have seen a substantial increase in cycling over the past 20 years (3.5% of commuters cycling to work in 1991, 8.2% in 2011). The third, Cambridge, is Britain's leading cycling city and has seen sustained high levels of cycling (28.2% of commuters cycling to work in 1991, 32.5% in 2011) (Census, 2013, Goodman, 2013).

In each of these three cities we again extracted cyclist and motorcyclist fatality data from STATS19, and identified the leading daily newspapers for each city (the Birmingham Evening Mail, the Bristol Post and the Cambridge Evening News). The Birmingham and Bristol newspapers were covered by the Lexis Library up to December 2014, but coverage only started in the late 1990s (February 1998 in Birmingham; July 1997 in Bristol). We therefore only searched for fatalities occurring during this same time period. The Cambridge newspaper was not covered in Lexis Library at all, and we therefore instead searched manually in this newspaper using microfilm archives in the British Library. These searches involved scanning the newspaper from cover to cover for all three-month periods subsequent to any fatality after January 1992. All articles identified this way contained the words used in our electronic search terms, i.e. would have been returned as ‘hits’ by an electronic search.

### 2.4 Statistical analysis

The pre-specified primary outcome for our study population of STATS19 fatalities was the proportion of fatalities receiving any media coverage within two years of fatality. For fatalities receiving any media coverage, we were also interested in the amount of coverage for each fatality. The secondary outcome was therefore the mean number of articles reported within two years per fatality. The upper time limit of two years was chosen to reflect the fact that a longer follow-up period was not available for the most recent fatalities.

We had an insufficient number of time points to undertake formal time series analysis, modelling how changes in cycling levels impacted changes in media coverage over time. We therefore instead investigated how our primary and secondary outcomes varied according to the year in which the fatality took place, examining whether media reporting of cyclist fatalities changed over time in line with the increase in total cycling levels. These analyses were stratified by city and by travel mode (bicycle versus motorcycle), and started with the calculation of raw proportions and means. We then proceeded to fit Poisson regression models with robust standard errors (Zou, 2004), in order to calculate risk ratios. We also used these Poisson models to test for interactions between year of fatality and travel mode–that is, whether trends differed over time between cyclist and motorcyclist fatalities. In the case of our secondary outcome, similar tests for interaction were performed after dichotomising the number of articles reported into 1–2 articles versus ≥3 articles.

In London, we additionally used multivariable Poisson regression models to assess whether location, age, sex, and strike mode were predictors of whether a given fatality received any media coverage. STATS19 had 100% complete data on all these characteristics except age, which was 98% complete. These missing data were imputed using multiple imputation by chained equations (25 imputations) under an assumption of missing at random. All analyses were conducted using Stata 13.1.

## 3 Results

### 3.1 Reporting of cyclist and motorcyclist fatalities over time in London

**Fig. 3:**
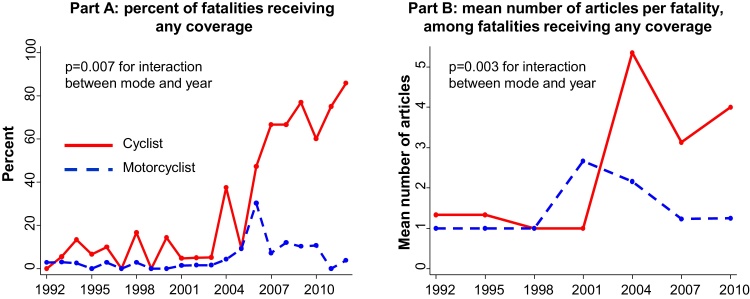
Proportion of cyclist and motorcyclist fatalities covered in the London Evening Standard, and average number of articles published among fatalities receiving any coverage, 1992–2012. Part A shows the proportion of fatalities covered among all 1218 fatalities. Part B shows the average number of articles published for each of the 135 fatalities who were covered at least once.

Across the study period, the annual number of cyclist fatalities in London remained relatively stable, at around 15 per year (Table 1). Given that the estimated daily number of cycle trips almost doubled (Fig. 2), this implies a reduced injury rate per cyclist over the time period. Although the total number of cyclist fatalities was fairly stable, the proportion covered in the London Evening Standard increased markedly, from 6% (3/51) in 1992–1994 to 78% (31/40) in 2010–2012 (Fig. 3A, Table 1). This translated into an adjusted risk ratio of 13 (Table 1) – i.e. after adjusting for the fatality's gender, age, the region of London and the strike mode, the likelihood of receiving any media coverage was around 13 times higher in 2010–2012 than in 1992–1994. This change was highly significant (*p* < 0.001 for difference across the study years), and largely occurred after 2003. Thus the increase in media propensity to report cycling fatalities coincided with the period in which cycling in London also increased most rapidly (Fig. 2).

There was likewise strong evidence that the mean number of articles per fatality increased over the study period (*p* < 0.001), even when we only calculated this mean in relation to those fatalities that received at least some coverage (Fig. 3B). This latter effect was partly driven by eight individuals killed between 2004 and 2011 who were each covered in 10–31 separate articles, with much of this sustained coverage being accounted for by articles calling for increased road safety. Across the years studied, the number of cyclist-fatality headlines that featured calls for cycle safety improvements, or criticism of current infrastructure, rose from 1/14 in 1992–2003 (7% of unique articles) to 30/84 in 2004–2012 (36% of articles, *p* = 0.03 for difference).

The proportion of motorcyclist fatalities covered in the Evening Standard also varied across the study period, being higher in 2004–2009 than in other years (Fig. 3A, Table 1). The magnitude of this variation was smaller than that seen for cyclists, however, and the increase in the mid-2000s was not sustained. There was little change across the time period studied in the number of articles reported per motorcyclist fatality (Fig. 3B). For both of these two outcomes, tests for interaction between year and fatality mode provided strong evidence (0.003 ≤ *p* ≤ 0.007) that the pattern seen for cyclists differed from that seen for motorcyclists. This provides some evidence that the observed pattern for cycling cannot be attributed to changes across the study period in the reporting of road traffic crashes more generally.

### 3.2 Other predictors of fatality coverage in London

Table 2 shows the association between other individual and crash-related factors and whether a cyclist or motorcyclist fatality received any coverage. There was no evidence that any of these predictors of coverage differed between cyclists and motorcyclists (all *p* > 0.25 in tests for interaction), so we pooled data from cyclists and motorcyclists in the regression analyses in order to increase power (see Supplementary Tables S1 and S2 for stratified results). In minimally-adjusted analyses, there was evidence that fatalities among females and among younger individuals were more likely to be covered, as were fatalities occurring in more central parts of London. There was also a trend towards higher coverage of fatalities where the strike mode was a heavy goods vehicle. In mutually-adjusted analyses these effects were attenuated but marginally significant effects remained for gender, age and area of London.

### 3.3 Reporting of fatalities outside London

In all three non-London cities, the level of coverage of both cyclist and motorcyclist fatalities was uniformly high across the whole of the study period, including 100% coverage of cyclist fatalities in Bristol and Cambridge (see Table 3). This uniformly high coverage meant that there was limited scope for any increase in coverage and this, in combination with the smaller numbers of fatalities (reflecting smaller population sizes), meant that there was insufficient power to conduct meaningful comparisons across time. The number of articles reported per fatality covered in the press was likewise high in these other settings relative to London. Overall, therefore, the three smaller UK cities had a pattern of newspaper coverage that was comparable to that seen in the most recent years for cycling in London.

## 4 Discussion

In London, the number of cycling trips has doubled in the last 20 years. Over the same period, the number of cyclist fatalities covered in the largest London newspaper has increased ten-fold, even though the total number of cyclist fatalities has remained stable. The timing of this increase in media coverage of cyclist fatalities closely followed the timing of the fastest increase in the prevalence of cycling. The observation that the coverage of motorcyclist fatalities remained low throughout the study period provides some evidence that the changes observed for cycling did not simply reflect wider shifts in how the newspaper covered road traffic crashes. It therefore seems plausible that the change in coverage for cyclist fatalities was instead specifically related to cycling becoming more ‘newsworthy’ as cycling became more common and as promoting cycling became an increasingly prominent transport goal for London policy-makers (Greater London Authority, 2013). Finally, our analyses also suggest that this increased frequency of covering cycling fatalities may have been accompanied by an increase in the number of articles campaigning for improved conditions for cyclists.

### 4.1 Refining the dynamic causal theory of changes in cycling levels

These findings suggest a need to refine and extend the dynamic causal theory that motivated this research. Our starting point for this research was the balancing loop B1 (*more fatalities make more stories*; see Fig. 1). This loop hypothesised that more cyclists would lead to more cycling fatalities and therefore to more media reports of those fatalities, and that this in turn would reduce public perceptions of safety and so dampen cycling uptake. In London, contrary to the prediction of this feedback loop, we found that coverage of motorcyclist fatalities did not increase despite an increase (in the middle of the study period) in the absolute number of deaths. We also found that the coverage of cyclist fatalities did increase in London without any increase in the number of cyclists killed. By contrast, the three smaller cities had almost universal media coverage of cycling fatalities, and so coverage changed in line with the total number of fatalities as proposed by the balancing loop B1. Taken together, we consider that the loop B1 may be relevant in some settings, but our findings provide some evidence that this loop is insufficient to capture fully the complex and context-specific role of the media in shaping cycling trends.

**Fig. 4:**
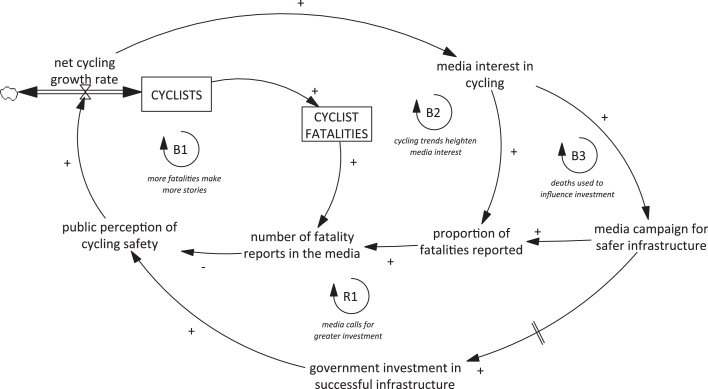
Updated causal loop diagram linking levels of cycling in a population and media representations of cycling. See Fig. 1 for notation. Loops labelled ‘R’ are ‘reinforcing loops’, which have the effect of amplifying the initial pattern of behaviour. The arrow shown with a double strikethrough denotes a time delay.

At least in London, we therefore propose that two further feedback loops are likely to be active (see Fig. 4). Firstly, a rapid increase in the uptake of cycling may lead to an increase in media interest even without an accompanying rise in cyclist fatalities (balancing loop B2 *cycling trends heighten media interest*). This leads to an increased likelihood of reporting fatalities in the media, again plausibly reducing public perception of cycling safety and acting as a hidden limit to the growth of cycling uptake. On a more positive note, if part of this increased reporting is tied to campaigns to improve cycling safety, then the media may in the longer term also play a role in influencing safety investment as part of a self-perpetuating reinforcing loop (reinforcing loop R1 *media calls for greater investment*).

Previous qualitative work in London has suggested a shift in recent years towards more media advocacy on behalf of cyclists (Aldred, 2013), and our findings also provide preliminary evidence of an increase in media campaigning for ‘safe cycling’ across the past two decades. Thus while the increased media coverage of cycling fatalities may have discouraged individuals from cycling, it may have influenced policy-maker behaviour in a different direction towards being more supportive of cycling. Further research could usefully investigate the ‘audience effect’ aspects of these loops, i.e. how individuals and policy-makers respectively understand and react to media coverage.

If media stories about fatalities are accompanied by repeated calls for greater government investment in cycling facilities, and if building these facilities successfully improves both objective and perceived safety, then this could lead to a more rapid uptake of cycling and further media interest. There would, however, be a delay between media campaigning and investment, hence the indication in Fig. 4 that this feedback loop would operate with a time lag. Moreover, this strategy may be undermined if media safety campaigns involve reigniting stories about fatalities (balancing loop B3 *deaths used to influence investment*). It could also be undermined if media campaigns focused primarily upon the need for individuals to cycle more cautiously, as opposed to calling for safety improvements to the wider cycling environment (Horton, 2007). We intend to explore this last point further in future qualitative work, which will provide an in-depth analysis of the content of the media fatality reports, and how this content may have changed over time.

In London, our analysis suggests that the balancing loops B2 and B3 are probably at present the strongest of these feedbacks. The results from other smaller cities, however, hint this may not be the case in all settings. In the data we examined, these cities appear already to be at or near ‘saturation point’ such that every cycling fatality is reported. This may well reflect the fact that absolute numbers of fatalities are smaller in these cities, and therefore any given fatality more newsworthy. It is interesting to note that the saturation apparent in our data applies across all three cities, even though their low numbers of fatalities arise from somewhat different processes (a moderate population but very low cycling levels in Birmingham versus a to small population but high cycling levels in Cambridge). In the context of such saturation, the balancing loop B1 is likely to be most active, in that any increase or decrease in the absolute numbers of fatalities would be expected to translate into increases or decreases in levels of media coverage. There would also be the potential for reinforcing loop R1 to operate in these cities, depending on the predominant media discourse.

### 4.2 Implications for encouraging cycling and improving cycling safety

The need for policies that proactively improve cycling safety is reinforced by the potential for media coverage of cycling deaths to undermine policy objectives to increase the number of people cycling. In large cities, where increased cycling levels may lead to dramatic increases in coverage per fatality (as in London), there is a particularly urgent need to reduce cycling risks to the much lower levels seen in higher-cycling contexts (e.g. by improving cycling infrastructure). Moreover, the changes in media reporting practices observed in our data also highlight that objective cycling risk is not the only influence on subjective risk perceptions. Such influences on risk perceptions are therefore also worthy of independent attention. By offering a more nuanced understanding of the complex, dynamic relationships between cycling numbers, fatalities and responses by the media, we hope to assist in creating more effective policies to meet the objectives of increasing both cycling levels and cycling safety. We also believe that similar approaches could usefully be applied to risk perceptions stemming from personal experience rather than from the media. For example, previous research has indicated the importance of experienced near-miss incidents to perceived safety (Joshi et al., 2001), so action to improve driver behaviour might be another means of mitigating the loops identified in Fig. 4.

Finally, although our primary focus was on changes in the reporting of fatalities over time, we also found some evidence that the London newspaper we examined was more likely to cover cyclist or motorcyclist fatalities in more central parts of London. This might be because the newspaper's readers (and the newspaper's journalists) are more likely to live in, work in or visit these central areas, thus illustrating the frequent role that ‘proximity’ plays when determining what is newsworthy (Conley and Lamble, 2006, Caple and Bednarek, 2013). Fatalities among women were also more likely to be covered, which may be related to women being perceived as more ‘vulnerable’ as road users than men, despite men's generally higher injury risks (Mindell et al., 2012). It is interesting to speculate how this might differentially impact on take-up. Cycling in London continues to be male-dominated (Aldred et al., 2015) and it is possible that women may be disproportionately influenced by hearing about other women's deaths while cycling. Thus the balancing loop identified here could perhaps also operate to reinforce the existing inequalities in take-up.

### 4.3 Study limitations and directions for future research

Strengths of our study include our innovative attempt to link individual police records of road traffic crashes to media coverage of those fatalities; our examination of a time period spanning two decades; and our use of motorcyclist fatalities as a control group for cyclists. One limitation is that we only examined one newspaper in each setting. Although the London Evening Standard is the oldest and largest daily newspaper in the London region, our findings may not generalise to its competitors or to alternative sources (e.g. online news). In addition, although the format of the Evening Standard has remained fairly stable over time (and has consistently been captured by LexisNexis), news-reading habits have changed considerably in the past 20 years. These changes in news-reading habits may mean that the total exposure of the public to coverage of cycling fatalities has increased to a greater or lesser degree than the increase documented in the Evening Standard. In addition, our study focussed only on the UK, and it is unclear how far the findings generalise to other parts of the world. It would therefore be a useful extension in future research to make comparisons of different media sources, as well as between international cities that have experienced substantial increases in cycling such as New York (NYC, 2013).

A further limitation of our study was its exclusive focus on newspaper reports of fatalities. One useful line of future research would be to assess how far our findings generalise to coverage of serious injuries among cyclists. This would be a particularly interesting extension in the smaller English cities examined, in order to investigate whether coverage of serious injuries is below ‘saturation point’ and may therefore have had scope to change over time (i.e. in line with loop B2). Another useful extension would be to set newspaper reports of fatalities in the context of all newspaper reporting on cycling over the time period (including pro-cycling ‘positive’ stories). One previous study which did this using Melbourne and Sydney newspapers found that a high number of cyclist fatality and injury stories were observed in both 1998–1999 and 2008–2009, but that overall the ratio of ‘negative’ to ‘positive’ cycling stories decreased over the decade examined (Rissel et al., 2010). If such effects have also been operating in London, it is possible that these counter-balance to some extent the balancing feedback loops shown in Fig. 4.

Another limitation of our study is that it only provides empirical evidence regarding the first part of the systems model shown in Fig. 4, i.e. the link between cycling levels and media reporting. In other areas, there appears to be well-developed evidence that the media has complex agenda-setting and framing effects on public opinion, as well as contributing in this way to setting a wider civic policy agenda (McCombs and Reynolds, 2009). This wider role was echoed during our previous qualitative research, during which a wide range of stakeholders hypothesised a number of relationships between media coverage and public perceptions of safety (Macmillan and Woodcock, 2013). It would be highly valuable in future research to complement the evidence presented in this paper with further empirical evidence regarding these other hypothesised relationships, and therefore by to extend further our understanding of the system dynamics of urban cycling. It would likewise be valuable to examine more closely the direct and indirect impacts of media coverage of cycling deaths on policy decisions and investment in cycling safety infrastructure and campaigns.

Finally, the present study was limited in largely focussing on ‘whether’ a fatality was covered in local newspapers, with less attention given to the content of that coverage. In other words, we largely focussed on the first role of the media discussed by McCombs’ agenda-setting theory of the media (McCombs, 2013) (which issues are worthy of public consideration) rather than the second role (how those issues are discussed). We intend to address this second role in a future qualitative paper which will examine how the Evening Standard covers cyclist fatalities; whether this has changed over time; and how this compares to coverage of motorcyclist fatalities or coverage in the three other English cities. This further qualitative research will also examine more fully the extent to which media coverage of cyclist fatalities may be instrumental to wider media campaigns to improve cycling infrastructure or cycling policy. Bringing the quantitative and qualitative research together, we hope to provide further insights into how local media may facilitate or hinder efforts to promote cycling and to improve cycling safety, including further refining our causal theory.

## Conflict of interest

None declared.

## Figures and Tables

**Table 1:** Proportion of cyclist and motorcyclist fatalities receiving any London Evening Standard newspaper coverage by year, 1992–2012 (*N* = 1218).

Year	Cyclist fatalities			Motorcyclist fatalities		
	*N* fatalities	% Receiving any coverage	Adjusted risk ratio (95% CI)	*N* fatalities	% Receiving any coverage	Adjusted risk ratio (95% CI)
1992–1994	51	6%	1***	109	3%	1***
1995–1997	47	6%	1.09 (0.23, 5.13)	91	1%	0.36 (0.04, 3.24)
1998–2000	36	11%	1.89 (0.45, 7.95)	143	1%	0.24 (0.03, 2.12)
2001–2003	60	5%	0.85 (0.18, 4.04)	201	1%	0.52 (0.10, 2.63)
2004–2006	48	31%	5.31 (1.64, 17.24)	134	14%	4.89 (1.44, 16.62)
2007–2009	43	74%	12.65 (4.16, 38.52)	130	10%	3.55 (0.99, 12.66)
2010–2012	40	78%	13.18 (4.33, 40.06)	85	5%	1.72 (0.38, 7.68)

CI: confidence interval. Risk ratios were almost identical in unadjusted analyses (see Supplementary Tables S1 and S2). See also Fig. 3A for a graphical representation of these data.

*** *p* < 0.001 for heterogeneity, adjusting for the fatality's gender, age, the region of London and the strike mode.

**Table 2:** Demographic and geographic predictors of any London Evening Standard newspaper coverage among cyclist and motorcyclist fatalities 1992–2012 (*N* = 1218).

	Cyclist fatalities (*N* = 325)		Motorcyclist fatalities (*N* = 893)		Risk ratio of any coverage, combining cyclist and motorcyclist fatalities (95% CI)	
	*N* fatalities	% covered	*N* fatalities	% covered	Minimally-adjusted	Mutually-adjusted
Region						
Outer	126	20%	530	4%	1**	1*
Inner	136	29%	292	7%	1.43 (1.02, 2.00)	1.38 (0.95, 1.99)
Central	63	41%	71	4%	1.73 (1.22, 2.45)	1.53 (1.02, 2.28)
Gender						
Male	232	23%	865	5%	1**	1†
Female	93	40%	28	14%	1.50 (1.10, 2.03)	1.35 (0.98, 1.87)
Age						
<18	38	24%	55	7%	1.19 (0.78, 1.83)	1.70 (1.02, 2.81)
18–34	118	35%	536	4%	1*	1†
35–64	123	30%	276	6%	1.07 (0.79, 1.45)	1.20 (0.89, 1.63)
≥65	37	8%	8	0%	0.40 (0.14, 1.13)	0.54 (0.19, 1.52)
Strike mode						
Car/Taxi	112	19%	422	5%	1†	1
Heavy goods vehicle	140	33%	91	7%	1.49 (1.06, 2.09)	1.30 (0.91, 1.87)
Other/no other vehicle	73	33%	380	4%	1.11 (0.76, 1.62)	1.07 (0.73, 1.56)

† *p* < 0.1, *p < 0.05, **p < 0.01, using tests for heterogeneity for gender and strike mode, and tests for trend for region and age. Minimally-adjusted analyses adjust for year, travel mode and the interaction between year and travel mode. Mutually-adjusted analysis additionally adjust for all variables displayed in the table.

**Table 3:** Predictors of any newspaper coverage among cyclist and motorcyclist fatalities in three non-London comparison cities, in comparison to London before versus after the large increase in cycling.

	Population in 2001	Cyclist fatalities			Motorcyclist fatalities		
		*N* fatalities	% fatalities receiving any coverage	Mean no. articles per fatality covered	*N* fatalities	% fatalities receiving any coverage	Mean no. articles per fatality covered
Birmingham (1998–2012)	977,000	16	81%	3.4	52	79%	4.2
Bristol (1997–2012)	381,000	11	100%	2.9	36	83%	3.0
Cambridge (1992–2012)	109,000	13	100%	n/a	6	100%	n/a

London (1992–2002)	7,172,000	175	7%	1.2	481	15%	1.7
London (2003–2012)	7,172,000	150	53%	4.3	412	9%	1.7

n/a: number of articles per fatality not available in Cambridge as manual rather than electronic searching was used, and searches were not made across a full two years following each fatality.
