# Supplementary material for: Trends in local newspaper reporting of London cyclist fatalities 1992-2012: the role of the media in shaping the systems dynamics of cycling
Source: Accid Anal Prev. 2016 Jan;86:137–45. doi: 10.1016/j.aap.2015.10.016 (PMC4682170; doi:10.1016/j.aap.2015.10.016)
Supplement: Supplementary file 1 [file mmc1.docx]

SUPPLEMENTARY MATERIAL

Figure S1: Annual number of cyclist and motorcyclist fatalities recorded in STATS19 on London roads, 1992-2012

**
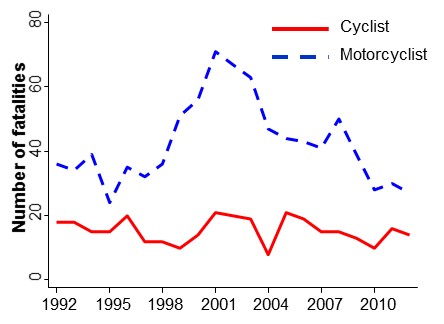
**

Table S1: Predictors of any London Evening Standard newspaper coverage among cyclist fatalities 1992-2012 (N=325)

|  |  | **N** | **% receiving** | **Risk ratio (95% CI)** | |
| --- | --- | --- | --- | --- | --- |
|  |  | **fatalities** | **any coverage** | **Minimally-adjusted** | **Mutually-adjusted** |
| **Year** | **1992-1994** | 51 | 6% | 1*** | 1*** |
|  | **1995-1997** | 47 | 6% | 1.09 (0.23, 5.13) | 1.11 (0.23, 5.33) |
|  | **1998-2000** | 36 | 11% | 1.89 (0.45, 7.95) | 1.72 (0.41, 7.14) |
|  | **2001-2003** | 60 | 5% | 0.85 (0.18, 4.04) | 0.78 (0.17, 3.67) |
|  | **2004-2006** | 48 | 31% | 5.31 (1.64, 17.24) | 5.45 (1.66, 17.90) |
|  | **2007-2009** | 43 | 74% | 12.65 (4.16, 38.52) | 11.39 (3.78, 34.27) |
|  | **2010-2012** | 40 | 78% | 13.18 (4.33, 40.06) | 12.88 (4.19, 39.59) |
| **Region** | **Outer** | 126 | 20% | 1** | 1 |
|  | **Inner** | 136 | 29% | 1.35 (0.95, 1.92) | 1.17 (0.79, 1.74) |
|  | **Central** | 63 | 41% | 1.78 (1.25, 2.51) | 1.39 (0.93, 2.08) |
| **Gender** | **Male** | 232 | 23% | 1** | 1† |
|  | **Female** | 93 | 40% | 1.44 (1.12, 1.87) | 1.28 (1.00, 1.64) |
| **Age** | **<18** | 38 | 24% | 1.00 (0.72, 1.41) | 1.68 (1.00, 2.82) |
|  | **18-34** | 118 | 35% | 1** | 1* |
|  | **35-64** | 123 | 30% | 0.91 (0.67, 1.22) | 1.08 (0.79, 1.48) |
|  | **≥65** | 37 | 8% | 0.39 (0.14, 1.08) | 0.53 (0.19, 1.52) |
| **Strike** | **Car/Taxi** | 112 | 19% | 1* | 1 |
| **mode** | **Heavy Goods** | 140 | 33% | 1.81 (1.25, 2.63) | 1.62 (1.04, 2.50) |
|  | **Light Goods** | 23 | 30% | 1.79 (0.89, 3.58) | 1.93 (0.94, 3.94) |
|  | **Bus** | 20 | 40% | 1.86 (1.15, 2.99) | 1.63 (0.97, 2.73) |
|  | **Other/No other vehicle** | 30 | 30% | 1.51 (0.94, 2.44) | 1.38 (0.81, 2.38) |

†p <0.1, *p < 0.05,**p < 0.01,***p < 0.001, using tests for heterogeneity for gender and strike mode, and tests for trend for year, region and age. Minimally-adjusted analyses adjust for year group. Mutually-adjusted analysis additionally adjust for all variables displayed in the table.

Table S2: Predictors of any London Evening Standard newspaper coverage among motorcyclist fatalities 1992-2012 (N=893)

|  |  | **N** | **% receiving** | **Risk ratio (95% CI)** | |
| --- | --- | --- | --- | --- | --- |
|  |  | **fatalities** | **any coverage** | **Minimally-adjusted** | **Mutually-adjusted** |
| **Year** | **1992-1997** | 200 | 2% | 1*** | 1*** |
|  | **1998-2003** | 344 | 1% | 0.58 (0.15, 2.30) | 0.58 (0.14, 2.31) |
|  | **2004-2006** | 134 | 14% | 7.09 (2.47, 20.39) | 7.03 (2.37, 20.82) |
|  | **2007-2009** | 130 | 10% | 5.00 (1.67, 15.01) | 5.11 (1.67, 15.68) |
|  | **2010-2012** | 85 | 5% | 2.35 (0.60, 9.20) | 2.46 (0.63, 9.68) |
| **Region** | **Outer** | 530 | 4% | 1* | 1* |
|  | **Inner** | 292 | 7% | 1.88 (1.05, 3.37) | 1.82 (1.00, 3.30) |
|  | **Central** | 71 | 4% | 1.59 (0.50, 5.09) | 1.70 (0.54, 5.31) |
| **Gender** | **Male** | 865 | 5% | 1* | 1† |
|  | **Female** | 28 | 14% | 3.01 (1.21, 7.50) | 2.72 (0.99, 7.44) |
| **Age** | **<34** | 591 | 5% | 1 | 1 |
|  | **≥35** | 284 | 6% | 1.12 (0.62, 2.01) | 1.05 (0.58, 1.91) |
| **Strike** | **Car/Taxi** | 422 | 5% | 1 | 1 |
| **mode** | **Heavy Goods** | 91 | 7% | 1.18 (0.51, 2.76) | 1.15 (0.49, 2.74) |
|  | **Light Goods** | 69 | 4% | 0.79 (0.25, 2.47) | 0.76 (0.24, 2.42) |
|  | **Bus** | 35 | 6% | 1.10 (0.28, 4.35) | 1.04 (0.27, 4.04) |
|  | **Other/No other vehicle** | 276 | 4% | 0.71 (0.35, 1.41) | 0.73 (0.36, 1.48) |

See notes to Supplementary Table S1. Some year bands were pooled for motorcycling due to small numerators.
